# Effects of β-Carotene and Its Cleavage Products in Primary Pneumocyte Type II Cells

**DOI:** 10.3390/antiox6020037

**Published:** 2017-05-21

**Authors:** Cornelia Haider, Franziska Ferk, Ekramije Bojaxhi, Giuseppe Martano, Hanno Stutz, Nikolaus Bresgen, Siegfried Knasmüller, Avdulla Alija, Peter M. Eckl

**Affiliations:** 1 Department of Cell Biology and Physiology, University of Salzburg, Hellbrunnerstr. 34, Salzburg A-A-5020, Austria; haider.cornelia@gmail.com (C.H.); ekramije.bojaxhi@stud.sbg.ac.at (E.B.); nikolaus.bresgen@sbg.ac.at (N.B.); 2 Institute of Cancer Research, Department of Internal Medicine 1, Medical University of Borschkegasse 8a, Vienna A-1090, Austria; franziska.ferk@meduniwien.ac.at (F.F.); siegfried.knasmueller@meduniwien.ac.at (S.K.); 3 Department of Molecular Biology, University of Salzburg, Hellbrunnerstr. 34, Salzburg 5020, Austria; giuseppe.martano@hotmail.it (G.M.); hanno.stutz@sbg.ac.at (H.S.); 4 Department of Biology, University of Prishtina, Xhorxh Bush, n.n., Prishtina 10000, Kosova; avdulla.alija@uni-pr.edu

**Keywords:** β-carotene, β-carotene cleavage products, apo-8′carotenal, dimethoxy-naphthoquinone, pneumocytes, Comet assay, micronuclei, apoptosis

## Abstract

β-Carotene has been shown to increase the risk of developing lung cancer in smokers and asbestos workers in two large scale trails, the Beta-Carotene and Retinol Efficacy Trial (CARET) and the Alpha-Tocopherol Beta-carotene Cancer Prevention Trial (ATBC). Based on this observation, it was proposed that genotoxic oxidative breakdown products may cause this effect. In support of this assumption, increased levels of sister chromatid exchanges, micronuclei, and chromosomal aberrations were found in primary hepatocyte cultures treated with a mixture of cleavage products (CPs) and the major product apo-8′carotenal. However, because these findings cannot directly be transferred to the lung due to the exceptional biotransformation capacity of the liver, potential genotoxic and cytotoxic effects of β-carotene under oxidative stress and its CPs were investigated in primary pneumocyte type II cells. The results indicate that increased concentrations of β-carotene in the presence of the redox cycling quinone dimethoxynaphthoquinone (DMNQ) exhibit a cytotoxic potential, as evidenced by an increase of apoptotic cells and loss of cell density at concentrations > 10 µM. On the other hand, the analysis of micronucleated cells gave no clear picture due to the cytotoxicity related reduction of mitotic cells. Last, although CPs induced significant levels of DNA strand breaks even at concentrations ≥ 1 µM and 5 µM, respectively, β-carotene in the presence of DMNQ did not cause DNA damage. Instead, β-carotene appeared to act as an antioxidant. These findings are in contrast with what was demonstrated for primary hepatocytes and may reflect different sensitivities to and different metabolism of β-carotene in the two cell types.

## 1. Introduction

β-Carotene (BC) has been demonstrated to possess antioxidant activity in vitro by scavenging peroxyl radicals (in particular lipid peroxyl radicals, nitrogen dioxide-, thiyl-, and sulfonyl-radicals) by quenching singlet oxygen and by inhibiting lipid peroxidation [1,2,3,4,5,6]. Diseases associated with increased oxidative stress could therefore be prevented by its uptake, and it has been shown that the increased uptake of carotenoids or fruits and vegetables as primary source of carotenoids reduces the risk to develop cardiovascular diseases [7,8,9], age-dependent macula degeneration [10], cataract formation [11,12], and different types of cancer [13] such as mouth, pharynx, larynx, esophagus, stomach, colon, rectum, bladder, and cervix [14,15,16,17]. However, in two major chemoprevention trials (the Alpha-Tocopherol Beta-Carotene Cancer Prevention Study and the Beta-Carotene and RETinol Efficacy Trial), the incidence of cancer and death from coronary artery disease was increased after β-carotene supplementation in both cigarette smokers and asbestos workers [18,19,20]. It was therefore suggested by Wang and Russell [21] that β-carotene metabolites are responsible for the carcinogenic response, and Sommerburg et al. [22] were able to demonstrate that β-carotene is degraded in the culture medium via oxidants released by activated polymorphonuclear leukocytes. By applying hypochlorous acid to β-carotene as a model for neutrophil-derived degradation, they further proved the formation of volatile short-chain cleavage products (CPs) such as β-cyclocitral, β-ionone, 5,6-epoxy-β-ionone, dihydroactinidiolide, and 4-oxo-β-ionone, as well as long-chain CPs such as apo-8′-carotenal and apo-12′-carotenal [22]. These CPs have been demonstrated to modify respiratory burst, to induce apoptosis of human neutrophils [23], and to induce oxidative stress in vitro by impairing mitochondrial respiration [24,25].

Based on this observation, both a cleavage product mixture generated by hypochlorite bleaching of β-carotene and one of the major carotenals contained—apo-8′-carotenal—were tested for their genotoxic potential in the primary hepatocyte assay, both in the presence and absence of oxidative stress. These investigations demonstrated a dose-dependent genotoxic potential of the CPs, which was further enhanced in the presence of oxidative stress by hypoxia/reoxygenation or 2,3-dimethoxy-1,4-naphthoquinone (DMNQ) application. This genotoxic potential was not accompanied by cytotoxicity [26,27]. However, cytotoxicity appeared when hepatocytes were not proliferatively stimulated and is characterized by significantly increased rates of necrosis and apoptosis [28]. Yurtcu et al. [29] further showed significantly increased levels of DNA damage, apoptosis, and necrosis in HepG_2_ cells after the application of the plasma peak β-carotene concentration of 8 µM.

These findings indicate that β-carotene cleavage products are most likely responsible for the increased lung cancer risk observed in chemoprevention trials. However, there is a lack of information on the sensitivity of the putative target cells in the lung, namely alveolar type II cells. Alveolar type II cells have a central role in the maintenance of normal lung function, reaction to injury, and response to specific toxins. They express phase I and phase II biotransformation enzymes—particularly cytochrome P450-dependent mono-oxygenases [30]—and thus are potential targets for many inhaled materials, and at the same time represent the relevant cells for the evaluation of a mutagenic and carcinogenic potential of specific agents [31]. Therefore, the goal of this investigation was the evaluation of the cyto- and genotoxic potential of β-carotene and its cleavage products in primary rat pneumocytes.

## 2. Materials and Methods

### 2.1. Materials

Percoll was purchased from GE Healthcare, Vienna, Austria. RPMI 1640 liquid medium with stable l-glutamine was obtained from PAA, Pasching, Austria. Fetal calf serum (FCS), penicillin/streptomycin was purchased from Medpro, Vienna, Austria. DMNQ (CAS No. 6956-96-3, 99% purity) was purchased from ENZO Life sciences. THF (tetrahydrofuran, CAS No. 109-99-9, ≥99.9% purity) was from Sigma-Aldrich (http://www.sigmaaldrich.com/austria.html). β-Carotene (CAS No. 7235-40-7, ≥97% purity) was purchased from Calbiochem, Darmstadt, Germany. Trypsin was from SIGMA, Vienna, Austria. Plastic culture dishes were obtained from Greiner BioOne, Linz Austria and Sarstedt, Vienna, Austria. The mouse monoclonal antibody MAb 3C9 (Anti-ATP-binding cassette sub-family A member 3 antibody (3C9) recognizing P180 lamellar body protein was obtained from Abcam (http://www.abcam.com).

Low melting point agarose and normal melting point agarose (LMA and NMA) were obtained from Gibco (Paisley, UK). Inorganic salts, dimethyl sulfoxide (DMSO, CAS No. 67-68-5, ≥99% purity), ethidium bromide, Trizma base and trypan blue were purchased from Sigma-Aldrich (Steinheim, Germany).

### 2.2. Preparation of β-Carotene Cleavage Products (CPs)

The generation of CPs was performed as described by Siems et al. [24] and Sommerburg et al. [22] with slight modifications. For degradation, β-carotene was dissolved in methanol (CAS No. 67-56-1, ≥99.9% purity, Merck (http://www.merckmillipore.com) containing 2% (*v*/*v*) trichloromethane (CAS No. 67-66-3, 99–99.4% purity, Merck Millipore, http://www.merckmillipore.com) to achieve sufficient solubility of the carotenoid. Chemical destruction of β-carotene was done by bleaching with hypochlorite by adding NaClO (CAS No. 7681-52-9, 6–14% active chlorine basis, FLUKA http://www.analytics-shop.com/de/hersteller/fluka.html) in a 100-fold concentration relative to the carotenoid. The samples reacted at room temperature and in daylight for 10 min. The applied concentrations are given as µM of β-carotene used for degradation.

### 2.3. Animals

Female Fischer 344 rats weighing approximately 100 g were obtained from Harlan–Winkelman, (www.harlan.com) Germany. The animals were kept in a temperature (20–24 °C)- and humidity (55 ± 10%)-controlled room with a 12 h light–dark cycle. Food and water was provided ad libitum.

### 2.4. Methods

#### 2.4.1. Isolation and Culture of Pneumocyte Type II Cells

Pneumocyte type II cells were isolated following the procedure of Richards et al. [32] with some modifications reported by De Boeck et al. [31]. Procedures were performed in compliance with the national animal experimentation law 2012, permissions No. BMWFW-66.012/0016-WF/II/3b/2014 and BMWFW-66.012/0037-WF/V/3b/2016. In short, after extended anesthesia and death of the animal, the chest was opened and the trachea cannulated with an affiliated syringe allowing the ventilation of the lung. A catheter was then inserted into the right ventricle of the heart and the lungs washed with a 0.15 M NaCl solution. Thereafter the lung was perfused via the trachea with 0.25% trypsin (freshly prepared) in digestion buffer (142 mM NaCl, 6.7 mM KCl, 10 mM Hepes, 1.29 mM MgSO_4_, 89 mM CaCl_2_, and 1 mg/mL glucose adjusted to pH 7.4) for 20 min at 37 °C. The tissue was chopped and shaken, and FCS was applied to stop the enzyme reaction. After filtration via two polyamide nets (150 µM and 30 µM mash width), the cell suspension was separated on a discontinuous Percoll gradient (light gradient 1.040, heavy gradient 1.089). Cells having sedimented between the two gradients were collected and plated in a 90 mm Petri dish for 1 h at 37 °C to allow remaining macrophages to attach to the plate. Pneumocytes were collected, vitality was determined by the trypan blue exclusion technique, and cells were plated at a density of 2.5 × 10^5^ cells per 35 mm petri dish. Cells were used for experiments after five days in culture.

#### 2.4.2. Characterisation of Pneumocyte Type II Cells

**Figure 1:**
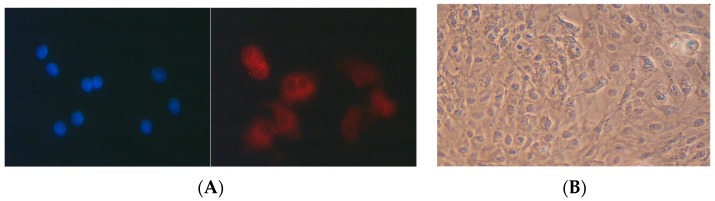
(**A**) Immunocytochemical detection of the lamellar body membrane protein in five day old cultures of pneumocyte type II cells—counterstaining with DAPI; (**B**) phase contrast micrograph of proliferating pneumocytes after five days in culture—arrows indicate metaphases.

In order to determine whether primary pneumocytes retain their characteristic features during primary culture, the expression of the marker protein lamellar body membrane protein was analyzed immunocytochemically after up to five days of primary culture. Even after five days in primary culture (Figure 1A), there is expression of the protein indicating that the phenotype of type II pneumocytes is retained. After five days of primary culture, pneumocytes were treated with the carotenoids. At this time, the cultures were almost confluent (Figure 1B) and characterized by proliferating cells.

#### 2.4.3. Treatment

CPs were dissolved in DMSO, while β-carotene was dissolved in tetrahydrofuran (THF). Stock solutions of the desired compounds were diluted in RPMI medium to final concentrations of 0.1, 1, 5, 10 µM apo-8′-carotenal and 0.1, 1, 10, 50 µM β-carotene. Solutions were prepared freshly prior to experiments. The concentration of the solvent was not exceeding 0.5% in the medium, a concentration which is not toxic to the cells as demonstrated by different authors [33,34,35]. The solvent controls themselves were 0.5% (THF) and 1% (DMSO) solutions.

Cells were incubated with the medium containing increasing concentrations of CPs or β-carotene for three hours under standard conditions in the incubator. For the experiments to evaluate the potential effects of oxidative stress on β-carotene the redox-cycling quinone DMNQ was additionally applied to the cells at a concentration of 40 µM which has been shown to cause significant cyto- and genotoxic effects in primary hepatocytes [27]. After three hours, the cells were washed twice with fresh medium to remove the applied substances, fresh medium was added and the cells returned to the incubator.

#### 2.4.4. Fixation, Staining and Cytogenetic Analysis

The cells were fixed in the dishes with methanol:glacial acetic acid (3:1) for 15 min, briefly rinsed with distilled water, and air dried. The fixed cells were stained with the fluorescent dye DAPI (4′,6-diamidino-2-phenylindol, 0.2 µg/mL McIlvaine citric acid-Na_2_HPO_4_ buffer, pH = 7) for 30 min, washed with McIlvaine buffer for 2 min, briefly rinsed with distilled water and mounted in glycerol. To determine the mitotic indices and the frequencies of micronucleated, apoptotic and necrotic cells 1000 cells per dish were analysed under a fluorescence microscope (Leitz Aristoplan) as described previously [36,37]. Cell densities as a measure for detached dead cells were evaluated by counting the number of adherent cells/visual field.

#### 2.4.5. Comet Assay

Cells were incubated with BC (0.10–10.0 µM) for 3 h (37 °C), and DMNQ (40 µM) was applied as oxidative agent in the experiments.

The cells were detached from Petri dishes by trypsinization (10 min). Subsequently, the cells were washed twice with phosphate buffered saline (PBS) and placed on agarose coated slides (10^5^ per slides). Acute toxic effects were monitored with the trypan blue dye-exclusion technique [38], and only cultures in which the cell viability was ≥80% were analyzed for DNA migration. After lysis and electrophoresis (20 min, 300 mA, 25 V, at 4 °C, pH > 13), the gels were stained with ethidium bromide (20 µg/mL).

The experiments were carried out according to the international guidelines for Comet assays published by Tice et al. [39] and Burlinson et al. [40]. For each experimental point, three cultures were made in parallel, and 50 cells were evaluated from each culture. The DNA damage was determined with a computer aided system (http://www.lucia.cz/en/front-page/lucia-comet-assay).

#### 2.4.6. Statistical Analysis

Data normal distribution was examined using the Shapiro-Wilks test. The statistical significance of pairwise comparisons was determined by applying Student’s double-sided independent samples t-test and the paired samples t-test (dependent variables). For multiple comparisons of normally distributed data ANOVA and the Welch test were applied using Levenes-test to check variance homogeneity and Tukey (homogenous variances) or Tamhane T2 (variances not homogeneous) post-hoc testing. The Kruskal–Wallis H Test and Mann-Whitney U tests were used for multiple comparisons of not normally distributed data. Statistical analysis was performed by employing the IBM SPSS Statistics software package (version 23).

In the single cell gel electrophoresis assays, the means and SD of % DNA in the comet tails of the nuclei from the different treatment groups were calculated. Comparisons of groups were done by one-way ANOVA followed by the Dunn’s multiple comparisons post-test. For all comparisons, *p* values ≤ 0.05 were considered as significant. Statistical analyses were performed using Graphpad Prism 4.0 (Graphpad Software, San Diego, CA, USA).

## 3. Results

### 3.1. Effects of CPs on Primary Pneumocytes

#### 3.1.1. Cytotoxicity

**Figure 2:**
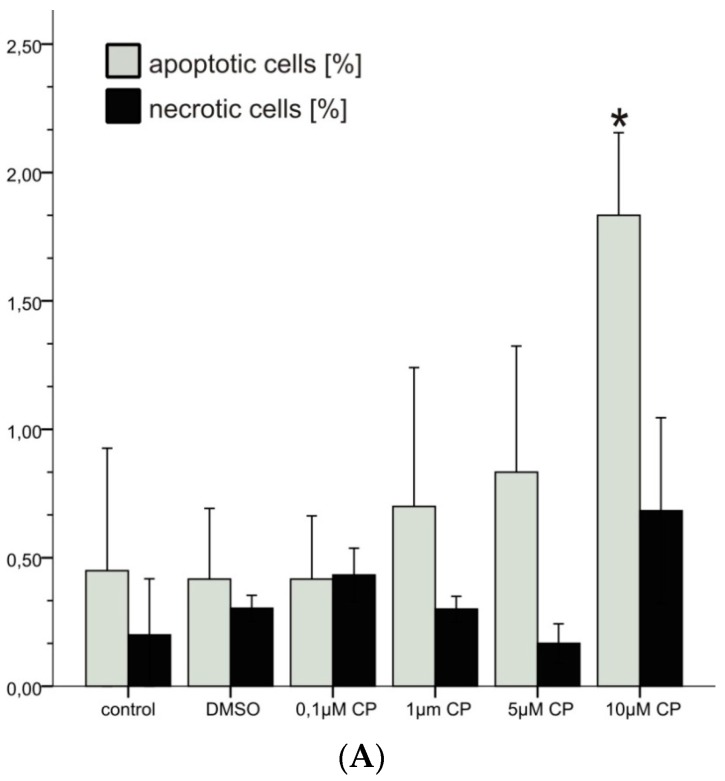

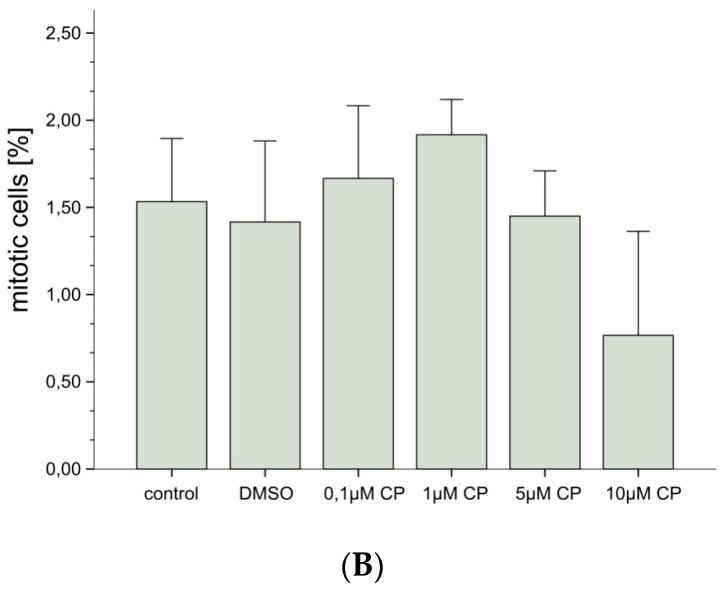
(**A**) Frequencies of apoptotic and necrotic cells and (**B**) frequencies of mitotic cells in primary pneumocyte cultures incubated with different concentrations of CPs; *: *p* < 0.05 compared to the DMSO control; *N* = 3.

Figure 2A shows the dose response of both apoptosis and necrosis induction by CPs (prepared by NaClO degradation). Up to a concentration of 5 µM there is no significant increase of apoptotic cells, although there is a trend to higher rates. At a concentration of 10 µM CPs, a significant increase (*p* < 0.05) of apoptotic cells is observed. The increased levels of apoptotic cells at the highest CPs concentration applied are accompanied by a trend to lower proliferation rates as indicated by the mitotic indices (Figure 2B). Due to high variations between the experiments, necrotic cells were not significantly elevated, even at a concentration of 10 µM CPs.

In these experiments, micronucleated cells were also evaluated. However, no significant changes were found up to a concentration of 10 µM (data not shown).

#### 3.1.2. Genotoxicity

##### Comet Assay

**Figure 3:**
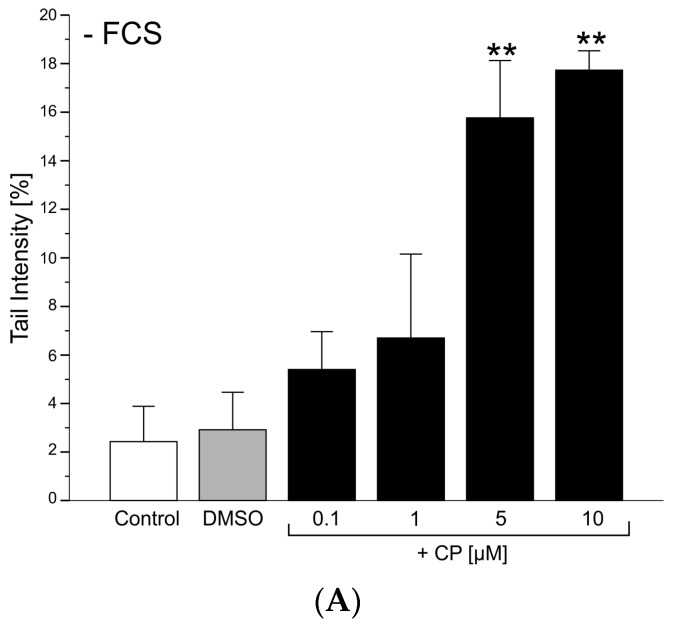

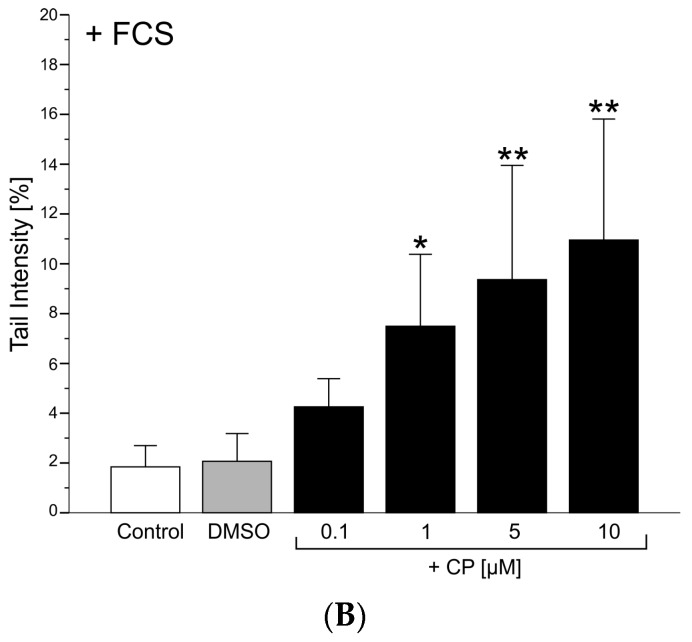
Effects of CPs on DNA-damage in primary cultures of rat pneumocytes; (**A**) treatment in the absence of FCS; (**B**) treatment in the presence of FCS; *: *p* < 0.05; **: *p* < 0.005 compared to the DMSO control (ANOVA, Tuckey post-hoc testing); *N* = 3.

In order to test whether the presence of fetal calf serum (FCS) in the culture medium might scavenge and thus detoxify part of the CPs, the experiments were carried out in the presence and absence of serum (Figure 3A,B). The results of the Comet assay clearly indicate that CPs induce a dose dependent increase of DNA damage in primary pneumocytes irrespective of the presence or absence of FCS. In cultures without FCS, there is a trend to slightly higher damages compared to serum supplemented cultures. Nevertheless CPs induced significant increases of DNA damage in serum supplemented cultures (Figure 3B) at concentrations ≥ 1 µM, while significant increases in serum-free cultures (Figure 3A) were observed at concentrations ≥ 5 µM. When regression analyses were applied to the data, it turned out that a linear model fits the data best. The slope of the regression curve was in fact steeper in the absence of FCS indicating that serum components may at least partly contribute to detoxification by binding of CPs. However, the statistical comparison of both treatments revealed no significant differences.

### 3.2. Effects of β-Carotene in the Presence of Oxidative Stress

#### 3.2.1. Cytotoxicity

Table 1 lists the effects of the relevant controls (THF, DMNQ, and 50 µM β-carotene) on the levels of mitotic, apoptotic, and necrotic cells. Not included are the results of the negative control (cell culture medium) because it is not relevant for the statistical comparison. The results of the negative control were: mitotic index: 2.6 ± 0.68; apoptotic cells: 0.42 ± 0.21; necrotic cells: 0.12 ± 0.10. In order to test for statistical differences between all controls (including the negative control), ANOVA was applied and revealed no statistical significance.

When primary pneumocytes were treated with increasing concentrations of BC dissolved in THF, neither the percentage of apoptosis or necrosis nor the mitotic indices showed any statistically significant changes even at the highest concentration of 50 µM (Table 1).

**Figure 4:**
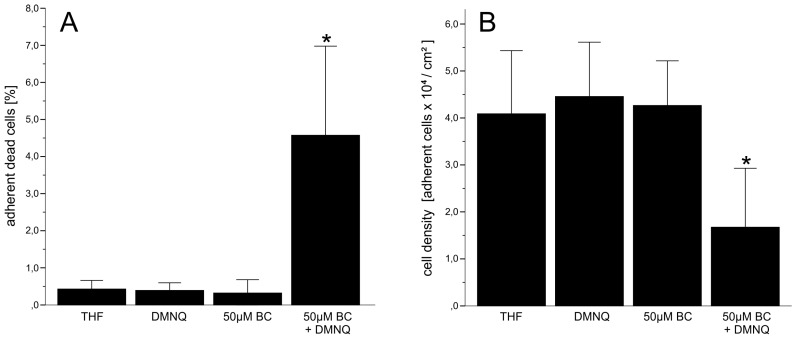
(**A**) Adherent dead cells and (**B**) changes of cell density in pneumocyte type II cell cultures treated with different concentrations of BC in the presence of 5 µM DMNQ. Data represent the means ± SD of at least 3 independent experiments; *: *p* < 0.05 compared to all controls.

In case of co-exposure with the redox cycling quinone DMNQ, a different behavior was experienced. Up to 10 µM, there was no significant difference to the controls (data not shown). The percentage of apoptotic and necrotic cells became significant at a concentration of 50 µM BC. At this concentration, a significant decrease of mitotic cells also occurred. This effect was accompanied by a significant increase of total cell death (apoptotic and necrotic cells; Figure 4A) and a significant reduction of the cell density (Figure 4B) indicating loss of cells due to increasing toxicity.

#### 3.2.2. Genotoxicity

BC dissolved in THF did not induce the formation of micronucleated pneumocytes. Instead, it caused a significant reduction (*p* < 0.05) at the highest concentration tested (50 µM). Since this reduction was not accompanied by a lower percentage of mitotic cells, this effect can eventually be attributed to the antigenotoxic potential of BC as described by different authors [41,42,43,44].

On the other hand, when β-carotene was applied in the presence of oxidative stress induced by DMNQ, a level of micronucleated cells (2.18 ± 1.07) comparable to the control (2.13 ± 0.48) was observed at a concentration of 50 µM. At the same concentration, a highly significant (*p* < 0.01) decrease of the proliferation rate as evidenced by the mitotic index (0.62 ± 0.37) was observed.

**Figure 5:**
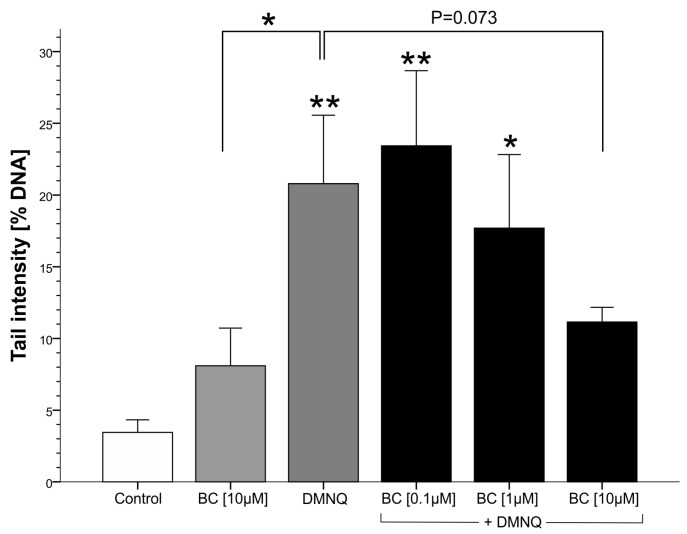
Effects of β-carotene under oxidative stress by DMNQ on DNA-migration in primary rat pneumocyte cultures. Per experimental point, three cultures were made in parallel, and from each culture 50 cells were evaluated. Bars indicate means ± SD of three independent experiments; *: *p* < 0.05 compared to the respective control; **: *p* < 0.01 compared to the control.

DMNQ is a compound that is considered to cause DNA single strand breaks via the formation of OH-radicals [45]. In fact, treatment with DMNQ led to a significant increase of DNA strand breaks, as evidenced by the Comet assay (Figure 5). This damage was expected to increase in the presence of β-carotene due to its cleavage and the release of genotoxic CPs. The results of the Comet assay, however, contradicted this assumption, since increasing β-carotene concentrations reduced DNA damage, and this reduction became almost significant (*p* = 0.073) at a β-carotene concentration of 10 μM, most likely indicating an antioxidant action of β-carotene.

## 4. Discussion

### 4.1. β-Carotene Cleavage Products

Primary pneumocytes react sensitively towards a mixture of CPs prepared by hypochlorite bleaching of β-carotene leading to significant increases of DNA damage already at concentrations as low as 1 μM even in the presence of serum in the growth medium as evidenced by the Comet assay (Figure 3).

The results of the Comet assay are representative for acute DNA damage, which may be repaired. Therefore, the formation of micronucleated cells as a measure of loss of genetic information due to chromosome breaks or disturbances of the mitotic spindle was further investigated. However, up to a concentration of 10 μM CPs, no significant increase of micronucleated cells was observed. This observation may indicate a lack of micronucleus formation. Still it has to be taken into consideration that the formation or appearance of micronucleated cells depends exclusively on cell division. A reduced proliferation rate will therefore influence the rate of detectable cells containing micronuclei. Such “hidden” damage can be estimated by standardizing the mitotic indices to 1%. In our example, the control level of micronucleated cells was 2.45% at a mitotic index of 1.53%.

Standardized to a mitotic index of 1% the percentage of micronucleated cells would be 1.6%. At a CPs concentration of 10 μM, the mitotic index was 0.76% and the percentage of micronucleated cells 2.36%. The standardized level would therefore be 3.1%, indicating an approximate doubling of micronucleated cells.

This indirect evidence for an increased formation of micronucleated cells is supported by the significantly increased percentage of apoptotic cells at a concentration of 10 μM CPs. Since apoptosis can be considered to be a mechanism for the safe removal of damaged cells, these results can also be interpreted in terms of cellular damage. While highly damaged cells will undergo apoptosis or even die due to a more acute mechanism of cell death as indicated by the elevated levels of necrotic cells, less damaged cells will have the capacity to proceed in the cell cycle and thus it can be expected that the numbers of micronucleated cells are lower.

### 4.2. β-Carotene under Oxidative Stress

Treatment with DMNQ in the presence of β-carotene did not cause genotoxicity as measured by the Comet assay. Instead, a β-carotene concentration-dependent decrease was observed (Figure 5). The results of the pneumocyte cytotoxicity and micronucleus assay partly confirm the findings obtained in the Comet assay because, up to a β-carotene concentration of 10 μM, no significant changes of necrotic and apoptotic cells as well as mitotic indices and micronucleated cells were observed, although the level of apoptotic cells is elevated at a concentration of 10 μM. However, when a β-carotene concentration of 50 μM was applied, significant changes were observed: a significant increase of apoptotic and necrotic cells, a highly significant reduction of the mitotic index, and a significant reduction of the cell density, indicating cell loss due to increased toxicity, while the percentage of micronucleated cells did not differ from control level.

As discussed above, this result may again depend on the reduced rate of cell proliferation masking a given genotoxic effect. Together with the significantly reduced cell density observed at a β-carotene concentration of 50 μM these results point to cytotoxicity as the major mechanism by which high concentrations of β-carotene under oxidative stress affect pneumocytes. Summarizing the following scenario can be envisaged:

β-Carotene is delivered to the lung via chylomicrons [46,47] and there taken up by macrophages [48]. Both the tar and gas phase of cigarette smoke contain free radicals and non-radical oxidants [49], and it is estimated that one puff contains approximately 10^14^ radicals [50]. While tar radicals are not highly reactive, gas phase radicals are generally more reactive [51]. Cigarette smoke, on the other hand, is chemotactic to neutrophils and macrophages and activates them [52,53,54], allowing gas phase radicals to interact with endogenous oxidants released from activated macrophages during oxidative burst [55]. As a consequence, particle-induced NO can react with the superoxide radical to form peroxynitrite [56], the homolysis of which generates the highly reactive OH-radical mediating tissue damage [57] via the initiation of lipid peroxidation. Similar to activated polymorphonuclear leukocytes [22], activated macrophages will cause the oxidative degradation of β-carotene and thus the formation of aldehydic and other breakdown products with different biological activities aggravating the impact of the oxidative burst. In fact, it has been demonstrated that β-carotene at a concentration that can be achieved in human plasma after chronic oral supplementation (5 µM) [58], and its metabolites were able to increase ^~^OH formation from H_2_O_2_ in the Fenton reaction and the addition of vitamin A and retinoic acid to lung epithelial cells co-cultured with activated neutrophils resulted in a significant increase of the level of oxidized purines [59], while the increase of oxidized purins was not significant after β-carotene treatment. These findings are in contrast to our findings with primary pneumocytes, as there was no indication of genotoxicity up to 10 µM. The question therefore arises as to whether DMNQ is an adequate model for oxidative stress in the lung. In this context, it has to be emphasized that DMNQ is an inducer of glutathione (GSH) [60], which is an essential element of the antioxidant defense [61,62]. GSH is the predominant scavenger of reactive oxygen species (ROS), particularly in the liver [62] and lung [63]. Under oxidative stress, the normal physiological ratio of ~100–1000 GSH:1 GSSG can be shifted toward the oxidized form, eventually even reaching an equimolar ratio [61,64]. GSSG is then exported out of the cells and metabolized [65]. Thus, relative levels of GSH and GSSG provide an efficient diagnostic option in judging the redox state of cells and hallmark oxidative stress, as demonstrated for several respiratory diseases and aging [62,66]. The lipid peroxidation product 4-hydroxynonenal is known to form adducts with GSH [67], and the immediate decrease of glutathione reported after smoking [54,68] can be attributed to this and other aldehydic lipid peroxidation products. While smoking significantly reduces cellular free glutathione (GSH) in experimental animals, especially in the lung, even after smoking periods as low as 30 days with exposures three times a day [69] DMNQ may eventually protect from oxidative damage. Therefore, the lack of an effect comparable to that found with primary hepatocytes [26] may relate to increased GSH levels in pneumocytes, and it can be assumed that co-cultivation of pneumocytes with alveolar macrophages or neutrophils, and subsequent activation will more realistically reflect the in vivo situation of smokers consuming β-carotene supplements.

## 5. Conclusions

In contrast to hepatocytes, pneumocytes are less sensitive toward CPs and BC in the presence of oxidative stress. Since pneumocytes are the putative target of β-carotene action, the obtained results could support the assumption that it is not causatively involved in adverse health effects because significant effects are obtained at concentrations that cannot be achieved even after chronic oral supplementation (i.e., 5 µM). Nevertheless, there is the possibility that DMNQ—as discussed—is an inappropriate inducer of oxidative stress in this particular cell type, and conclusive evidence may only be obtained by mimicking the situation in the lung during smoking.

## Figures and Tables

**Table 1:** Effects of β-carotene in the presence of DMNQ on mitotics, necrotic, and apoptotic cells in primary rat pneumocyte type II cells.

Treatment	Mitotic Cells (%) ^+^	Apoptotic Cells (%) ^++^	Necrotic Cells (%) ^+^
Control THF	2.95 ± 0.43 *	0.31 ± 0.24 (*p* = 0.077) ^1^	0.13 ± 0.15 **
Control DMNQ	2.49 ± 1.16 **	0.38 ± 0.19 (*p* = 0.084) ^1^	0.02 ± 0.04 **
Control 50 µM BC	2.51 ± 0.89 *	0.27 ± 0.27 (*p* = 0.074) ^1^	0.06 ± 0.09 **
50 µM BC + DMNQ	0.56 ± 0.12	3.91 ± 2.37	0.66 ± 0.19

Values are the mean ± SD of at least 4 independent experiments. ^+^ *p* < 0.05; ^++^ *p* < 0.005 for the observed differences between the percentages of mitotic cells (ANOVA, Welch test) or apoptotic and necrotic cells (Kruskal-Wallis H Test). * *p* < 0.05, ** *p* < 0.005 compared to the combined treatment (BC + DMNQ); Mann-Whitney U test (mitotic, necrotic cells). ^1^ Tamhane T2 post-hoc testing.
